# Relationship Between Participative Decision-Making Within an Organization and Employees’ Cognitive Flexibility, Creativity, and Voice Behavior

**DOI:** 10.3390/bs15010051

**Published:** 2025-01-08

**Authors:** Sun-Hee Kwon, Jeong-Sik Kim

**Affiliations:** 1 Department of Global Economics, College of Global Trade and Industry, Daejin University, Pochon-si 11159, Republic of Korea; sunnyk@daejin.ac.kr; 2 Department of Business Administration, College of Global Trade and Industry, Daejin University, Pochon-si 11159, Republic of Korea

**Keywords:** decision-making style, participative decision-making, cognitive flexibility, creativity, voice behavior

## Abstract

The existing literature predominantly examines the direct effects of participative decision-making, often overlooking the mechanisms and processes that mediate or moderate its outcomes. This study addresses this gap by investigating the impact of participative decision-making on employees’ cognitive flexibility, creativity, and voice behavior. Specific contradictions and gaps in prior research are highlighted, particularly the limited understanding of how these variables interact. This study is grounded in self-determination theory and incorporates a research model that examines these relationships through the mediating role of cognitive flexibility. Data were collected from 310 employees in South Korean firms and analyzed using structural equation modeling, providing robust empirical evidence. Key findings demonstrate that participative decision-making significantly enhances cognitive flexibility, which, in turn, mediates its impact on creativity and voice behavior. Direct effects were more pronounced for creativity, while cognitive flexibility played a stronger mediating role in fostering voice behavior. These results underscore the theoretical and practical importance of participative decision-making in fostering innovation and adaptability within organizations. Practical implications for managers and policymakers include fostering a participative culture to enhance employee creativity and voice behavior. Finally, this study discusses limitations, such as reliance on self-reported data, and provides directions for future research.

## 1. Introduction

Participative decision-making (PDM) has emerged as an essential practice for fostering collaboration, enhancing employee engagement, and driving innovation in modern organizations. By involving employees in decision-making processes, PDM builds trust, promotes autonomy, and improves communication, all of which are fundamental to achieving both individual and organizational success (Kim et al., 2022; Valverde-Moreno et al., 2021). While substantial research has examined the direct effects of PDM on outcomes such as job satisfaction and organizational performance (Goñi-Legaz & Ollo-López, 2017; Kim & Chah, 2013), there is limited understanding of the mechanisms that connect PDM to individual-level behaviors like creativity and voice behavior.

Creativity, defined as the ability to generate novel and useful ideas, is widely recognized as a cornerstone of organizational success in today’s rapidly changing markets (Runco & Jaeger, 2012). According to self-determination theory, environments that foster autonomy enable individuals to realize their creative potential by reducing external control and enhancing intrinsic motivation (Amabile, 1996; Runco & Beghetto, 2019). PDM helps create such environments by encouraging active employee participation in decision-making, which facilitates the generation of innovative solutions through the exchange of diverse perspectives. Recent studies have expanded the understanding of creativity by emphasizing its process-oriented nature, involving iterative exploration and refinement of ideas (Green et al., 2023). Furthermore, Giancola et al. (2022) have demonstrated the importance of cognitive styles in creativity, noting that flexible thinking enhances individuals’ ability to solve problems through both divergent and convergent approaches. These findings highlight the critical role of organizational practices like PDM in nurturing creative outputs.

In addition to creativity, voice behavior is another key outcome of PDM. Defined as the discretionary expression of constructive suggestions or concerns aimed at improving organizational processes, voice behavior represents an essential avenue for innovation and continuous improvement (Morrison, 2011). PDM fosters psychological safety, empowering employees to voice their opinions without fear of reprisal (Detert & Burris, 2007). This openness encourages proactive communication, enabling organizations to tap into employee insights for ongoing development (Silla et al., 2020). In today’s knowledge-driven economies, voice behavior has become increasingly important for facilitating knowledge-sharing and accelerating innovation (Khalil et al., 2021). The interplay between PDM and voice behavior is particularly significant for promoting adaptability and resilience in dynamic and uncertain environments.

Cognitive flexibility, the capacity to adapt one’s thinking and behaviors to new or changing contexts, plays a pivotal mediating role in the relationship between PDM, creativity, and voice behavior (Ionescu, 2012; Walia, 2019). Participative practices expose employees to diverse perspectives and challenging ideas, requiring them to adjust their cognitive frameworks. This adaptability not only supports creativity by enabling the integration of novel information but also enhances voice behavior by empowering employees to articulate and defend alternative viewpoints (Chen et al., 2014; Feng et al., 2020). The research further shows that cognitive flexibility underpins innovative problem-solving by fostering mental agility and openness to new ideas (De Dreu et al., 2011). Moreover, Mones and Massonnié (2022) found that individuals with higher cognitive flexibility generate more innovative ideas and exhibit greater confidence in presenting them, particularly in collaborative environments.

Despite these advancements, several critical gaps remain in the literature on PDM. First, most prior studies have primarily focused on the direct effects of PDM, overlooking the mediating mechanisms that explain its influence on individual behaviors like creativity and voice behavior. Second, there is a lack of integrated models exploring the relative contributions of PDM’s direct and indirect effects. Third, the relationship between cognitive flexibility, creativity, and voice behavior remains underexplored, especially in the context of participative practices. This study seeks to address these gaps by developing a unified framework that examines both the direct and indirect effects of PDM through cognitive flexibility, providing a more holistic understanding of its psychological mechanisms.

The expected contributions of this study are threefold. First, PDM is anticipated to have a significant direct effect on creativity, highlighting its immediate impact on innovative thinking. Second, PDM’s influence on voice behavior is expected to operate more strongly through the mediating role of cognitive flexibility, as adaptability is critical for articulating and advocating alternative perspectives. Third, cognitive flexibility is hypothesized to serve as a key mechanism linking PDM to both creativity and voice behavior, underscoring its importance in fostering innovation and proactive communication. By addressing these gaps, this study aims to advance the theoretical understanding of PDM while offering practical strategies for organizations seeking to optimize their participative practices.

## 2. Theoretical Background and Hypotheses

### 2.1. Participative Decision-Making and Cognitive Flexibility

Participative decision-making refers to an environment in which all organizational members’ opinions and ideas are actively considered and incorporated into the decision-making process and information exchange (Arnold et al., 2000). This approach is grounded in trust among organizational members and provides them with opportunities to voice their opinions, which leads to positive impacts on the members and ultimately improves organizational performance (Goñi-Legaz & Ollo-López, 2017; Kim & Chah, 2013). Furthermore, participative decision-making significantly influences members during the process of carrying out tasks. Specifically, it allows members to encounter and consider diverse perspectives and ideas from others, enhancing their ability to address issues flexibly by examining decision-making matters from both their own and others’ viewpoints (Martin & Rubin, 1995; Silla et al., 2020; Zhou & George, 2001).

Importantly, this process is closely linked to the enhancement of cognitive flexibility, a crucial cognitive function that enables individuals to adapt their thinking and behavior in response to changing environments and situational demands (De Dreu et al., 2011). Cognitive flexibility involves the ability to shift attention between different tasks, perspectives, or problem-solving strategies, vital for effective decision-making and problem-solving in dynamic organizational contexts (Chen et al., 2014). Participative decision-making, by exposing members to a variety of perspectives and encouraging collaborative brainstorming, creates a fertile environment for the development of cognitive flexibility (Feng et al., 2020). Therefore, participative decision-making can be seen as a critical antecedent of better cognitive flexibility. Based on this, the following hypothesis can be proposed:

**Hypothesis** **1.** *Participative decision-making is positively correlated with members’ cognitive flexibility.*

### 2.2. Participative Decision-Making and Creativity

Traditional psychological research on creativity has largely focused on the traits of creative individuals. However, recent studies have moved beyond individual characteristics to consider group and organizational characteristics as well as the work environment (Amabile, 1996; Runco & Jaeger, 2012; Zhou & George, 2001). In particular, creativity within organizations is influenced by the environment and, in turn, impacts individuals. Therefore, a thorough analysis of the complex interactions between individual and organizational characteristics and the environment is necessary (Amabile, 1996).

Creativity, defined as the ability to generate novel and useful ideas, is a critical factor in organizational success, driving innovation and adaptability in rapidly changing markets (Runco & Jaeger, 2012). According to self-determination theory, members can exhibit higher levels of creativity when they are free from external control or constraints through autonomous job performance. Participative decision-making enhances creativity by allowing organizational members to autonomously present their opinions during the decision-making process and listen to others’ ideas (Amabile, 1996; Kim et al., 2022). This process not only empowers individuals but also fosters a collaborative environment where diverse perspectives can lead to innovative solutions.

The increased sense of self-determination fostered by participative decision-making encourages members to engage more actively in their tasks and acts as a significant facilitator of creativity. By creating an environment where members feel valued and autonomous, organizations can significantly boost the creative output of their teams (Zhou & George, 2001). Thus, participative decision-making is crucial in enhancing creativity within organizations, leading to a positive impact on overall organizational performance (Kim & Chah, 2013). Based on these discussions, the following hypothesis can be proposed:

**Hypothesis** **2.** *Participative decision-making is positively correlated with members’ creativity.*

### 2.3. Participative Decision-Making and Voice Behavior

According to Morrison (2011), voice behavior is a discretionary form of communication in which employees express ideas, suggestions, concerns, or opinions regarding job-related issues to improve the functioning of their organization or department. Voice behavior includes not only highlighting necessary improvements and suggestions but also openly expressing views or opinions about others’ actions or ideas (Van Dyne & LePine, 1998). Integrating previous findings, voice behavior can be seen as a proactive and initiative-driven action aimed at enhancing the functionality of the organization and department. It represents a significant, intentional behavior that encompasses both personal and others’ viewpoints.

Voice behavior, critical to fostering innovation and continuous improvement within organizations, is closely linked to the concept of psychological safety—where members feel secure in expressing their opinions without fear of negative consequences (Edmondson, 1999). Participative decision-making provides members with various opportunities within the decision-making process and acts as a facilitator for voice behavior by enabling free expression, grounded in a sense of psychological safety (Kim et al., 2022). When employees believe their opinions can influence organizational decisions, they are more likely to engage in voice behavior, fostering organizational trust and reducing the fear of retaliation (Detert & Burris, 2007).

Furthermore, participative decision-making enhances self-determination and autonomy, encouraging members to take a proactive interest in important job-related issues. This empowerment leads to the presentation of diverse opinions and ideas, thereby promoting more proactive voice behavior (Kim, 2022). By enabling employees to feel that their contributions are valued and impactful, participative decision-making significantly contributes to the cultivation of an environment where voice behavior thrives, ultimately leading to better organizational outcomes. Based on these discussions, the following hypothesis can be proposed:

**Hypothesis** **3.** *Participative decision-making is positively correlated with members’ voice behavior.*

### 2.4. Cognitive Flexibility, Creativity, and Voice Behavior

Cognitive flexibility is the ability to adapt one’s thinking to various situations, allowing individuals to quickly adjust to new information or changing environments and solve problems from multiple perspectives (Ionescu, 2012). This ability plays a critical role in problem-solving by enabling diverse approaches and seeking new solutions, which is particularly valued in today’s rapidly changing society. Cognitive flexibility significantly contributes to creativity, as individuals with high cognitive flexibility can swiftly adapt to new situations and attempt various approaches, thus favoring creative problem-solving (Walia, 2019). This capability is essential for solving complex problems, exploring new ideas without being constrained by existing thought patterns, and forming the foundation for creative thinking and behavior.

Theoretical models explaining the relationship between cognitive flexibility and creativity suggest that cognitive flexibility provides the mental adaptability necessary for generating new ideas and solving problems, facilitating the achievement of creative outcomes through various pathways (De Dreu et al., 2011). Additionally, cognitive flexibility involves adapting to new and unexpected environments by considering complex factors rather than individual responses (Chen et al., 2014). This adaptability encourages the application of new methods in response to changing situations, leading to more desirable solutions and positively impacting creativity by enabling problem-solving with novel approaches (Feng et al., 2020).

Empirical studies also support the positive relationship between cognitive flexibility and voice behavior. Mones and Massonnié (2022) found that individuals with high cognitive flexibility generate more ideas in dynamic environments. Ou et al. (2018) showed that cognitive flexibility can influence voice behavior, emphasizing its crucial role in fostering voice behavior. Based on these discussions, the following hypotheses can be formulated:

**Hypothesis** **4.** *Members’ cognitive flexibility is positively correlated with creativity.*

**Hypothesis** **5.** *Members’ cognitive flexibility is positively correlated with voice behavior.*

### 2.5. Mediating Role of Cognitive Flexibility Between Participative Decision-Making, Creativity, and Voice Behavior

Participative decision-making allows employees to actively engage in the organizational decision-making process, providing opportunities to present diverse opinions and ideas (Kim et al., 2022). This process instills a belief in employees that their ideas can contribute to the organization, playing a crucial role in fostering creativity (Amabile, 1996; Zhou & George, 2001). Participative decision-making enhances employees’ psychological safety, allowing them to propose various ideas freely without fear of failure, thus enhancing creativity (Edmondson, 1999). In terms of voice behavior, participative decision-making encourages employees to actively present their opinions by making them believe their views can be reflected in organizational decisions (Morrison, 2011). Moreover, participative decision-making promotes trust and psychological safety within the organization, enabling employees to engage in voice behavior freely without fear of retaliation.

Additionally, participative decision-making enhances cognitive flexibility, enabling employees to analyze problems from diverse perspectives and propose creative solutions (Chen et al., 2014; Martin & Rubin, 1995; Silla et al., 2020). Cognitive flexibility acts as a mediator by enabling members to reframe problems and consider alternative solutions, which can lead to more innovative and effective outcomes. Specifically, when members participate in decision-making processes, they are exposed to diverse perspectives and information, which challenges their existing cognitive frameworks. This challenge necessitates cognitive flexibility, allowing them to integrate new information and perspectives into their thinking processes. Consequently, this increased cognitive flexibility fosters higher levels of creativity by allowing members to break free from conventional thinking patterns. Additionally, this same cognitive flexibility enables members to engage more actively in voice behavior, as they feel more equipped to present and defend novel ideas and approaches.

This mediating effect is particularly potent in dynamic and complex organizational environments where the ability to adapt and innovate is crucial for success (Feng et al., 2020; Walia, 2019). Therefore, cognitive flexibility not only enhances the direct impact of participative decision-making on creativity and voice behavior but also serves as a crucial pathway through which these relationships are strengthened. Based on these discussions, the following hypotheses can be proposed:

**Hypothesis** **6.** *Members’ cognitive flexibility partially mediates the relationship between participative decision-making and their creativity.*

**Hypothesis** **7.** *Members’ c* *ognitive flexibility partially mediates the relationship between participative decision-making and their voice behavior.*

**Figure 1:**
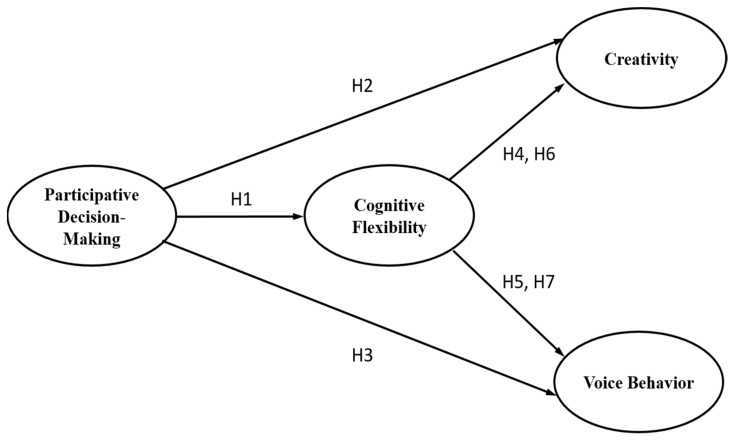
Proposed model.

The proposed model based on these hypotheses is shown in Figure 1.

## 3. Materials and Methods

### 3.1. Data Collection

Data collection for this study was conducted over six weeks, from 4 July to 14 August 2022, using a printed questionnaire survey targeting employees working in companies listed on the South Korean stock market. To ensure a representative sample, mid-sized companies or larger with assets exceeding KRW 500 billion were selected. A total of 18 companies from industries such as finance, services, information technology, electronics, distribution, chemicals, automobile, construction, and apparel participated in the survey. Prior approval was obtained from each organization.

The surveys were conducted in person by our research team. After contacting designated personnel within each company, we scheduled visits to distribute and collect the questionnaires. Employees were provided with printed questionnaires, which they completed on-site during the agreed-upon time slots. This direct method ensured consistent administration and allowed for real-time clarification of any participant questions.

A total of 360 questionnaires were distributed and collected during these visits, resulting in a preliminary response rate of 100%. After excluding incomplete or insincere responses, including those failing embedded attention checks, 310 valid responses were retained, yielding an effective response rate of 86.1%. Minimal missing data were resolved during the data cleaning process.

Participants consisted of 68.7% male respondents (213 individuals) and 31.3% female respondents (97 individuals). The average age was 38.6 years, with respondents distributed across various age groups: 5.2% in their 20 s, 54.2% in their 30 s, 35.5% in their 40 s, and 5.2% aged 50 or older. Educational backgrounds included high school graduates (1.9%), associate degree holders (3.9%), bachelor’s degree holders (64.5%), and those with graduate degrees (29.7%).

The industries represented included finance (5.8%), service (13.9%), information technology (8.4%), electronics (21.0%), distribution (10.6%), chemical (4.8%), automobile (4.5%), construction (11.6%), apparel (5.2%), and others (14.2%). Detailed demographic characteristics are presented in Table 1.

### 3.2. Measures

The measures utilized in this study were based on established scales, with all items rated on a five-point Likert scale (1 = strongly disagree, 5 = strongly agree). Participative decision-making was assessed using six items from Arnold et al. (2000), which included items such as “My supervisor listens to my work group’s ideas and suggestions”. Cognitive flexibility was measured with 10 items from Martin and Rubin (1995), including items like “I can find workable solutions to seemingly unsolvable problems”. Supervisors evaluated creativity using seven items from Zhou and George (2001), such as “My subordinate … comes up with new and practical ideas to improve performance”. Voice behavior was measured with six items from Van Dyne and LePine (1998), including “My subordinate … develops and makes recommendations concerning issues that affect this work group” (see Appendix A).

Cronbach’s alpha was utilized to assess the internal consistency of the scales for participative decision-making, cognitive flexibility, creativity, and voice behavior. Convergent validity was confirmed through factor loadings and average variance extracted (AVE) values, while discriminant validity was ensured by comparing the square root of the AVE with inter-construct correlations. By incorporating rigorous validation methods, this study provides robust and replicable insights into the relationships between participative decision-making, cognitive flexibility, creativity, and voice behavior.

## 4. Results

### 4.1. Validity and Reliability Analysis

To verify the validity of the data, confirmatory factor analysis (CFA) was conducted. The results demonstrated an acceptable fit to the data (*χ*2 = 1172.68, df = 554, comparative fit index (CFI) = 0.93, Tucker–Lewis Index (TLI) = 0.93, root mean square error of approximation (RMSEA) = 0.06) (see Table 2). All factor loadings for participative decision-making, cognitive flexibility, creativity, and voice behavior exceeded the threshold of 0.50, except for one item in cognitive flexibility. The average variance extracted (AVE) values for all constructs were above 0.50, confirming sufficient convergent validity (Hair et al., 2006). Discriminant validity was assessed by comparing the square roots of the AVE values with the correlation coefficients between variables. The square roots of the AVE values were consistently higher than the correlations with other variables, demonstrating discriminant validity (Fornell & Larcker, 1981). Composite reliability values for all constructs exceeded 0.70, further supporting the reliability of the measurements.

The results of the correlation analysis are shown in Table 3. There were significant correlations among all variables.

### 4.2. Hypotheses Testing

Structural equation modeling (SEM) was performed using Amos 23.0 to test the hypothesized model. The analysis revealed that the hypothesized model achieved acceptable fit indices (*χ*2 = 1182.66, CFI = 0.93, TLI = 0.93, RMSEA = 0.06). Competing models were also tested to evaluate the robustness of the hypothesized model. The competing models included variations where direct paths from participative decision-making to creativity and voice behavior were removed individually or simultaneously. The hypothesized model consistently showed lower chi-square values compared to the competing models, with all differences being statistically significant. Furthermore, the main fit indices (CFI, TLI, RMSEA) indicated that the hypothesized model provided the best overall fit. These findings confirm that the hypothesized model is the most favorable and reliable representation of the data. The detailed results of the CFA and SEM, including the fit indices and comparisons with competing models, are presented in Table 2, Table 3 and Table 4.

**Figure 2:**
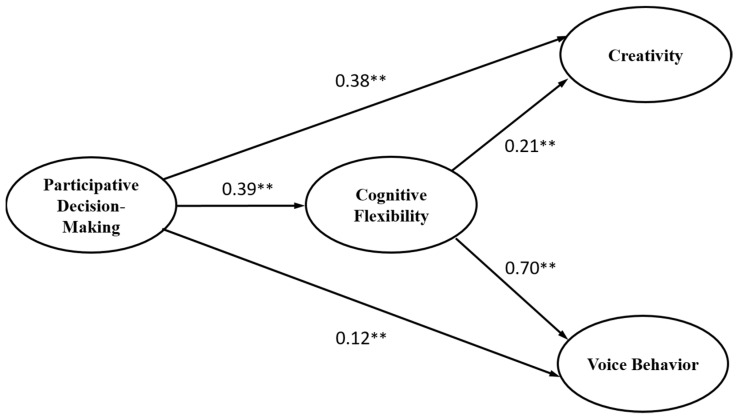
Analysis results of the hypothesized model. Note: ** *p* < 0.01.

The results obtained through the structural equation modeling analysis of the final research model are shown in Figure 2. Based on these analysis results, the verification of the research hypotheses is as follows. Participative decision-making had a significant positive relationship with cognitive flexibility, thereby supporting Hypothesis 1 (β = 0.39, *p* < 0.01). Participative decision-making had a significant positive relationship with creativity, thereby supporting Hypothesis 2 (β = 0.38, *p* < 0.01). Participative decision-making had a significant positive relationship with voice behavior, thereby supporting Hypothesis 3 (β = 0.12, *p* < 0.01). Cognitive flexibility had a significant positive relationship with creativity, thereby supporting Hypothesis 4 (β = 0.21, *p* < 0.01). Cognitive flexibility had a significant positive relationship with voice behavior, thereby supporting Hypothesis 5 (β = 0.70, *p* < 0.01).

Hypotheses 6 and 7 involved the mediating role of cognitive flexibility in the relationship between participative decision-making and creativity, as well as between participative decision-making and voice behavior. The significance of the mediating effect of cognitive flexibility was tested using the bootstrapping technique in AMOS. According to Shrout and Bolger (2002), significance is determined if 0 is not included in the confidence interval (CI) between the lower and upper bounds. As shown in Table 5, in the path from participative decision-making to creativity via cognitive flexibility, 0 was not included in the interval between the lower and upper bounds (95% CI = [0.03, 0.19]). Therefore, the mediating path from participative decision-making to creativity via cognitive flexibility was statistically significant (β = 0.08, *p* < 0.05), supporting Hypothesis 6. Similarly, for Hypothesis 7, the path from participative decision-making to voice behavior via cognitive flexibility did not include 0 in the interval between the lower and upper bounds (95% CI = [0.14, 0.33]). Therefore, the mediating path from participative decision-making to voice behavior via cognitive flexibility was also statistically significant (β = 0.27, *p* < 0.05), supporting Hypothesis 7.

## 5. Discussion

### 5.1. Summary

This study investigated the impact of participative decision-making (PDM) on employees’ cognitive flexibility, creativity, and voice behavior, with a particular focus on the mediating role of cognitive flexibility. The findings, grounded in the study’s hypotheses, contribute critical insights into how PDM shapes individual and organizational outcomes. This section contextualizes these findings within the broader literature, highlighting areas of agreement, divergence, and contribution to theoretical and practical understanding.

First, PDM significantly influenced both creativity and voice behavior. This aligns with self-determination theory, which posits that autonomy fosters intrinsic motivation and creativity (Deci & Ryan, 1985). In participative environments, employees are empowered to contribute innovative ideas, driven by a sense of psychological safety and ownership. These findings are consistent with Morrison’s (2011) assertion that psychological safety is a key enabler of voice behavior, as it minimizes fear of reprisal and encourages employees to engage in proactive communication. However, our findings extend previous research by illustrating how PDM not only motivates employees to express novel ideas but also fosters collaboration that directly supports creativity. While existing studies often highlight the direct effects of PDM on organizational performance (Goñi-Legaz & Ollo-López, 2017; Kim & Chah, 2013), this study enriches the understanding of PDM’s role in shaping individual contributions by emphasizing its capacity to balance autonomy with structured engagement.

Second, the results revealed that PDM significantly enhances cognitive flexibility, a finding that challenges traditional views of cognitive flexibility as a stable individual trait. This study demonstrates that external organizational practices, such as participative environments, can cultivate cognitive adaptability by exposing employees to diverse perspectives and challenging ideas. These findings resonate with Martin and Rubin’s (1995) work on cognitive flexibility, which emphasizes its role in generating solutions and adapting to changing demands. However, our results extend this literature by showing how PDM operationalizes these cognitive processes through active engagement and inclusivity. This broader interpretation suggests that cognitive flexibility is not merely an inherent capability but also a skill that organizations can strategically develop through participative practices. In doing so, this study addresses a critical gap in the literature by linking organizational strategies to individual adaptability, highlighting a novel pathway for enhancing employee cognitive capabilities.

Third, cognitive flexibility emerged as a significant antecedent of creativity and voice behavior. Employees with higher cognitive flexibility demonstrated a stronger ability to synthesize diverse perspectives and apply them to organizational challenges. This supports prior findings that cognitive flexibility facilitates creative thinking by enabling the exploration of alternative solutions (Stad et al., 2018) and enhances voice behavior by empowering employees to confidently present and advocate for constructive feedback (Chen et al., 2014). Importantly, this study revealed a nuanced divergence in how cognitive flexibility interacts with creativity versus voice behavior. Creativity benefited more directly from PDM, reflecting the immediate collaborative and decision-making autonomy fostered in participative environments. In contrast, voice behavior relied more heavily on cognitive flexibility, as employees navigating the social risks and complexities of proposing alternative viewpoints required adaptability and mental agility. This distinction underscores the psychological demands unique to each outcome and highlights the differential pathways through which PDM operates.

Finally, cognitive flexibility was identified as a critical mediator in the relationship between PDM and both creativity and voice behavior. While PDM directly influenced creativity more strongly, its effect on voice behavior was primarily mediated through cognitive flexibility. This nuanced finding reflects the dual mechanisms by which PDM enhances organizational outcomes. Participative practices directly foster creativity by providing an environment conducive to innovation, while the development of cognitive flexibility amplifies employees’ capacity to engage in voice behavior. These results align with prior research suggesting that voice behavior often entails navigating social complexities, requiring more adaptive psychological processes than creativity (Detert & Burris, 2007). By distinguishing these pathways, this study provides a deeper understanding of how PDM operates as a multifaceted organizational tool, capable of simultaneously driving innovation and proactive communication.

Together, these findings reinforce the theoretical framework proposed in this study and contribute to the broader literature on PDM, creativity, and voice behavior. By highlighting the mediating role of cognitive flexibility, this study bridges existing gaps in the literature and advances the understanding of how participative practices influence both cognitive and behavioral outcomes. The implications for practice are equally significant, as organizations can leverage PDM not only to directly inspire creativity but also to cultivate cognitive flexibility as a pathway for enhancing voice behavior. This dual strategy provides a comprehensive approach to fostering innovation and adaptability in dynamic organizational environments.

### 5.2. Implications for Theory and Practice

This study offers meaningful contributions to both theoretical development and practical application within the field of organizational behavior. By integrating cognitive flexibility into the participative decision-making (PDM) framework, the research provides a deeper understanding of the psychological mechanisms underlying participative practices. Unlike prior studies that predominantly focused on the direct effects of PDM on outcomes such as job satisfaction and performance, this study identifies cognitive flexibility as a critical mediator linking PDM to creativity and voice behavior. This finding expands existing theoretical frameworks by emphasizing the importance of cognitive processes in shaping employee adaptability and innovative contributions.

Theoretically, this study challenges traditional views that cognitive flexibility is an inherent individual trait, demonstrating instead that it can be cultivated through external organizational practices like PDM. This insight aligns with dynamic systems theories, which suggest that psychological traits are influenced by environmental factors. By showing that participative environments foster cognitive adaptability, this research advances the understanding of how organizational practices influence individual cognitive capabilities, bridging the gap between behavioral and cognitive outcomes in organizational settings. Furthermore, this study highlights the dual mechanisms of PDM’s influence, showing that creativity benefits more directly from participative practices, while voice behavior is enhanced through the mediating role of cognitive flexibility. This nuanced perspective adds depth to self-determination theory and complements theories of proactive behavior by providing a more comprehensive framework for understanding how participative practices drive employee innovation and communication.

Practically, the findings offer actionable strategies for managers and organizations. First, organizations should recognize PDM as more than a decision-making tool—it is a strategic mechanism for fostering engagement, creativity, and proactive communication. Managers should create structured opportunities for employee involvement, such as collaborative planning sessions, brainstorming workshops, and regular feedback forums. These participative initiatives not only enhance employees’ sense of belonging and responsibility but also stimulate innovative thinking and open communication.

Second, organizations should prioritize initiatives that build cognitive flexibility among employees. Training programs focused on adaptive thinking, problem-solving, and exposure to diverse perspectives can enhance employees’ mental agility, equipping them to navigate the complexities of modern organizational challenges. Leadership development programs should also emphasize participative leadership styles, ensuring that managers have the skills to involve employees effectively in decision-making processes. Tailoring PDM practices to employee readiness and task complexity will maximize their impact, fostering an environment where participative practices thrive.

Finally, organizations should cultivate a culture of inclusion and psychological safety. Employees are more likely to engage in creative and proactive behaviors when they feel their contributions are valued and respected. Managers should actively promote an environment where diverse perspectives are celebrated and employees are encouraged to challenge the status quo without fear of reprisal. By embedding these values into organizational culture, companies can harness the full potential of their workforce, enhancing both individual and collective adaptability and resilience.

In summary, this study contributes to the theoretical development of participative practices by elucidating the mediating role of cognitive flexibility and advancing our understanding of its dynamic interaction with PDM. From a practical standpoint, the findings underscore the importance of participative strategies that enhance both cognitive and behavioral outcomes, offering a roadmap for organizations seeking to foster innovation, engagement, and adaptability in an ever-changing business landscape.

### 5.3. Limitations and Suggestions for Future Research

Despite the meaningful insights provided by this study, several limitations must be acknowledged, which open up avenues for future research. Addressing these limitations will allow researchers to deepen our understanding of participative decision-making (PDM) and its influence on cognitive flexibility, creativity, and voice behavior.

First, the reliance on self-reported data for constructs such as PDM and cognitive flexibility may have introduced response biases. While supervisor evaluations were utilized to assess creativity and voice behavior, the potential for common method bias cannot be entirely eliminated. To enhance the robustness of future studies, researchers could adopt mixed-method approaches, such as combining qualitative interviews with quantitative surveys. These methods would provide richer insights and mitigate biases inherent in single-method studies. Moreover, longitudinal research designs should be considered to capture the dynamic and temporal nature of the relationships examined in this study, thereby addressing the limitation of cross-sectional data.

Second, this study focused exclusively on cognitive flexibility as a mediator between PDM and employee outcomes. While the results underscore its critical role, other potential mediators—such as intrinsic motivation, perceptions of fairness, and psychological empowerment—were not examined. Future research could incorporate these variables to develop a more comprehensive understanding of the mechanisms through which PDM influences creativity and voice behavior. For instance, intrinsic motivation may further illuminate how participative environments foster creativity, while perceptions of fairness could provide insights into how PDM promotes voice behavior. Additionally, exploring the interplay among multiple mediators could yield a nuanced understanding of the complex pathways linking PDM to organizational outcomes.

Third, this study was conducted exclusively in South Korean companies, which may limit the generalizability of the findings to other cultural contexts. South Korea’s collectivist culture and hierarchical organizational structures may have uniquely shaped the relationships observed in this study. For example, PDM in collectivist cultures may resonate more with employees who are accustomed to group-oriented decision-making. Conversely, in individualistic cultures, the effects of PDM on employee outcomes may differ due to varying expectations of autonomy and collaboration. Cross-cultural research could explore whether these findings are consistent across diverse cultural settings, offering valuable insights into how cultural norms and values influence the effectiveness of PDM.

Fourth, this study did not explicitly account for moderating variables that might influence the relationships examined. For example, job complexity, team dynamics, and leadership styles could affect how PDM impacts cognitive flexibility, creativity, and voice behavior. Including these moderators in future studies could enhance the explanatory power of the findings. For instance, in highly complex jobs, the role of PDM in fostering cognitive flexibility might be more pronounced. Similarly, team dynamics such as trust and cohesion could amplify or attenuate the effects of PDM on voice behavior. Understanding these moderating factors would enable the development of more tailored recommendations for organizations.

Finally, external environmental factors, such as market volatility, organizational size, or industry type, were not explicitly considered in this study. These factors could influence the effectiveness of PDM and its impact on employee outcomes. Future research could investigate how such external variables interact with PDM to shape organizational behaviors and performance, further refining the theoretical and practical implications of participative practices.

By addressing these limitations, future research can build on the foundation established by this study, advancing the theoretical and practical understanding of how PDM fosters innovation, adaptability, and proactive behaviors across diverse organizational contexts.

### 5.4. Conclusions

This study investigated the complex relationships among participative decision-making (PDM), cognitive flexibility, creativity, and voice behavior, with a focus on understanding the mediating role of cognitive flexibility. Through a combination of theoretical exploration and empirical analysis, this study provided robust evidence supporting the pivotal role of PDM in fostering key organizational behaviors.

The findings revealed that PDM not only directly influences creativity and voice behavior but also indirectly enhances these outcomes through its impact on cognitive flexibility. Participative environments encourage employees to engage with diverse perspectives, fostering a sense of autonomy and accountability that motivates creative thinking and proactive communication. These results extend the existing literature by identifying cognitive flexibility as a critical psychological mechanism that bridges participative practices and employee outcomes. This nuanced understanding sheds light on the dual pathways—direct and indirect—through which PDM operates, offering a comprehensive perspective on its organizational value.

The theoretical contributions of this study include expanding the understanding of PDM’s impact beyond surface-level outcomes by integrating cognitive, behavioral, and characteristic dimensions. By empirically validating the mediating role of cognitive flexibility, this study addresses a significant gap in the literature and provides a framework for exploring how participative practices shape employee behavior in dynamic organizational contexts.

From a practical standpoint, the findings underscore the importance of implementing PDM as a strategic tool for fostering an innovative and adaptable workforce. Organizations that prioritize participative practices can not only enhance creativity and voice behavior but also cultivate cognitive flexibility, which is essential for navigating uncertainty and driving organizational growth. Leaders are encouraged to adopt participative approaches by creating environments that value diversity, respect diverse viewpoints, and encourage open communication. Structured interventions, such as training programs to enhance adaptive thinking and leadership development initiatives, can further amplify the benefits of PDM.

Despite its significant contributions, this study is not without limitations. The reliance on self-reported data and the cross-sectional nature of the research highlight the need for future studies to adopt longitudinal designs and incorporate additional mediating and moderating variables. Furthermore, exploring cross-cultural variations in the effectiveness of PDM would provide valuable insights into its applicability across different organizational and cultural contexts.

In conclusion, this study offers a comprehensive understanding of how participative decision-making influences cognitive flexibility, creativity, and voice behavior, both directly and indirectly. By uncovering the psychological mechanisms that underpin these relationships, the research provides valuable guidance for organizations aiming to foster innovation, adaptability, and proactive communication. As the modern workplace becomes increasingly dynamic and complex, the strategic implementation of PDM presents a powerful pathway to achieving sustained organizational success.

## Figures and Tables

**Table 1:** Demographic characteristics of the respondents.

Variable	Category	Frequency	Percentage (%)
Gender	Women	213	68.7
	Men	97	31.3
Age	20–29	16	5.2
	30–39	168	54.2
	40–49	110	35.5
	Above 50	16	5.2
Educational Level	High School Graduate	6	1.9
	Vocational College Graduate	12	3.9
	University Graduate	200	64.5
	Graduate School Graduate	92	29.7
Industry	Finance	18	5.8
	Service	43	13.9
	Information Technology	26	8.4
	Electronics	65	21.0
	Distribution	33	10.6
	Chemical	15	4.8
	Automobile	14	4.5
	Construction	36	11.6
	Apparel	16	5.2
	Other	44	14.2

**Table 2:** Validity and reliability verification.

Construct	Item	Factor Loading	CR	AVE
Participative Decision-Making	1	0.57	0.86	0.60
	2	0.79		
	3	0.52		
	4	0.86		
	5	0.81		
	6	0.73		
Cognitive Flexibility	1	0.64	0.90	0.57
	2	0.74		
	3	0.76		
	4	0.62		
	5	0.62		
	6	0.79		
	7	0.73		
	8	0.80		
	9	0.40		
	10	0.74		
Creativity	1	0.89	0.98	0.80
	2	0.90		
	3	0.91		
	4	0.90		
	5	0.84		
	6	0.79		
	7	0.82		
	8	0.89		
	9	0.88		
	10	0.90		
	11	0.90		
	12	0.92		
	13	0.92		
Voice Behavior	1	0.76	0.92	0.70
	2	0.77		
	3	0.85		
	4	0.85		
	5	0.83		
	6	0.77		

Note: the factor loadings are standardized measurements.

**Table 3:** Correlation analysis results.

Construct	Mean	SD	1	2	3	4
Participative Decision-Making	3.61	0.81	(0.77)			
Cognitive Flexibility	3.83	0.61	0.37 **	(0.75)		
Creativity	3.47	0.89	0.47 **	0.34 **	(0.89)	
Voice Behavior	3.92	0.69	0.37 **	0.68 **	0.39 **	(0.83)

Notes: The diagonal values represent the square roots of the AVE values. Off-diagonal values are the correlation coefficients between constructs. ** *p* < 0.01.

**Table 4:** Comparison of hypothesized model and competing models.

	*χ*2	df	*χ*2/df	CFI	TLI	RMSEA	Δ*χ*2	Δdf
Hypothesized Model	1182.66 **	555	2.13	0.93	0.93	0.06	-	-
Competing Model 1	1220.08 **	556	2.19	0.93	0.92	0.06	37.42	+1
Competing Model 2	1188.21 **	556	2.14	0.93	0.93	0.06	5.55	+1
Competing Model 3	1223.73 **	557	2.26	0.93	0.92	0.06	41.07	+2

Note: Δ*χ*2 value is the difference in chi-square values between the competing model and the hypothesized model. ** *p* < 0.01.

**Table 5:** Results of the mediation analysis.

Pathway	Total Effect	Direct Effect	Indirect Effect	95% CI	
Participative Decision-Making → Cognitive Flexibility → Creativity	0.46	0.38	0.08	Lower	Upper
				0.03	0.19
Participative Decision-Making → Cognitive Flexibility → Voice Behavior	0.39	0.12	0.27	Lower	Upper
				0.14	0.33

## Data Availability

The data presented in this study are available on request from the corresponding author due to privacy or ethical reasons.

## Appendix A

**Table A1:** Questionnaires and sources.

Variable	Questionnaire	Source
Participative decision-making (six items)	My supervisor encourages work group members to express ideas and suggestions.	Arnold et al. (2000)
	My supervisor listens to my work group’s ideas and suggestions.	
	My supervisor uses my work group’s suggestions to make decisions that affect us.	
	My supervisor gives all work group members a chance to voice their opinions.	
	My supervisor considers my work group’s ideas when he/she disagrees with them.	
	My supervisor doesn’t make decisions that are based only on his/her own ideas.	
Cognitive flexibility (ten items)	I can communicate an idea in many different ways.	Martin and Rubin (1995)
	I can find workable solutions to seemingly unsolvable problems.	
	I have choices when deciding how to behave.	
	I am willing to work at creative solutions to problems.	
	In any given situation, I am able to act appropriately.	
	My behavior is a result of conscious decisions that I make.	
	I have many possible ways of behaving in any given situation.	
	I don’t have difficulty using my knowledge on a given topic in real life situations.	
	I am willing to listen and consider alternatives for handling a problem.	
	I have the self-confidence necessary to try different ways of behaving.	
Creativity (seven items)	My subordinate…suggests new ways to achieve goals or objectives.	Zhou and George (2001)
	My subordinate…comes up with new and practical ideas to improve performance.	
	My subordinate…searches out new technologies, processes, techniques, and/or product ideas.	
	My subordinate…is a good source of creative ideas.	
	My subordinate…exhibits creativity on the job when given the opportunity to.	
	My subordinate…develops adequate plans and schedules for the implementation of new ideas.	
	My subordinate…often has a fresh approach to problems.	
Voice behavior (six items)	My subordinate...develops and makes recommendations concerning issues that affect this work group.	Van Dyne and LePine (1998)
	My subordinate...speaks up and encourages others in this group to get involved in issues that affect the group.	
	My subordinate...communicates his/her opinions about work issues to others in this group even if his/her opinion is different and others in the group disagree with him/her.	
	My subordinate...keeps well informed about issues where his/her opinion might be useful to this work group.	
	My subordinate...gets involved in issues that affect the quality of work life here in this group.	
	My subordinate...speaks up in this group with ideas for new projects or changes in procedures.	
